# Hemodynamic discordance and rarity claims in a congenital ebsteinoid triad: a call for clarification

**DOI:** 10.1186/s43044-026-00801-z

**Published:** 2026-11-11

**Authors:** Mohammad Omar Tunio, Muhammad Aslam, Yashwini Sawlani, Salman Irshad Abbasi, Muhammad Amir Gadhi

**Affiliations:** Chandka Medical College, Larkana, Pakistan

To the Editor,

We read with great interest the case report by Karuru et al. describing an adolescent with an Ebsteinoid tricuspid valve anomaly, a giant right atrial aneurysm, and a secundum atrial septal defect (ASD) [1]. The authors are to be commended for the detailed multimodality imaging and the technically thoughtful surgical approach, particularly the preservation of conduction tissue during right atrial reduction atrioplasty. The reassuring one-year clinical outcome adds useful follow-up to the limited literature on this unusual congenital combination. We wish to raise several points that may further strengthen the haemodynamic interpretation and educational value of the report.

The report describes a peak tricuspid regurgitation (TR) Doppler velocity of 3.39 m/s, corresponding to a peak trans-tricuspid systolic pressure gradient of 46 mmHg. Right-heart catheterisation (RHC), in turn, reported right atrial (RA) mean pressure of 8 mmHg, right ventricular (RV) pressure of 32/6 mmHg, pulmonary artery (PA) pressure of 28/12 mmHg (mean 18 mmHg), and pulmonary vascular resistance of 1.1 Wood units·m² [1]. These measurements are related but are not directly interchangeable. The peak TR gradient represents the systolic pressure difference between the RV and RA, estimated using the simplified Bernoulli Eq. (4 V²). Echocardiographically estimated RV systolic pressure (RVSP) is obtained by adding estimated RA pressure to the TR gradient, provided there is no relevant RV outflow tract or pulmonary valve obstruction. Accordingly, the reported 46 mmHg is the TR gradient, not by itself the RVSP or PA systolic pressure. Importantly, the report does provide an invasive RV systolic pressure of 32 mmHg. The difference between this catheter-derived value and the Doppler-derived TR gradient merits clarification, including whether the echocardiographic and catheter measurements were obtained under comparable haemodynamic conditions, what RA pressure was estimated by echocardiography, and whether the figure caption’s description of an “RVSP gradient” is intended to denote the TR gradient alone or an RVSP estimate. The catheter-derived mean RA pressure of 8 mmHg should not automatically be substituted for an echocardiographically estimated RA pressure. These details would help readers interpret the apparent Doppler–catheterisation discordance. The 2025 American Society of Echocardiography recommendations provide an updated framework for comprehensive right-heart assessment [2]. As these recommendations address adults and the reported patient was 15 years old, they are cited here for general Doppler principles rather than as age-specific paediatric guidance.

The direction of the regurgitant jet also merits clarification. The case report’s main text describes an eccentric-posterior jet entering the aneurysmal RA, whereas the Fig. 4 caption describes flow directed towards the interatrial septum [1]. These descriptions may not be mutually exclusive if the jet is dynamic or has multiple components, with an initial posterior trajectory followed by redirection or wall-hugging flow along the aneurysmal atrial wall. A Coanda-type effect is one possible explanation for such a pattern, but it remains a hypothesis unless demonstrated by the available colour Doppler views. In a markedly enlarged and geometrically distorted atrium, jet direction and continuous-wave Doppler alignment may vary with imaging plane and acquisition. The authors could clarify whether the two descriptions refer to different portions or views of the same jet and, if possible, indicate its direction relative to the Doppler beam. Without direct supporting measurements, jet-vector effects and altered atrial compliance should be presented as possible mechanisms rather than established explanations for the pressure discrepancy.

The original report distinguishes the decision to omit catheter-based ventriculography or right-heart angiography from the performance of RHC for pressure and pulmonary vascular measurements [1]. These are complementary rather than contradictory decisions. Echocardiography and cardiac CT can provide detailed anatomical information, whereas RHC provides invasive haemodynamic measurements when these are needed to resolve uncertainty or inform operative planning. Accordingly, the distinction is not between invasive and non-invasive assessment in general, but between invasive structural/angiographic imaging and invasive haemodynamic assessment. Making this distinction explicit would avoid any implication that detailed non-invasive anatomical imaging makes invasive haemodynamic assessment redundant.

The description of this congenital combination as exceptionally rare should be appropriately qualified. Tsujii et al. reported a giant right atrial aneurysm associated with isolated displacement of the anterior tricuspid leaflet, a particularly relevant anatomical comparator, although that report does not describe the same complete triad of Ebsteinoid tricuspid abnormality, giant right atrial aneurysm, and secundum ASD [3]. Ashraf et al. also provide a relevant comparison involving an Ebstein anomaly variant with anterior leaflet displacement [4]. These reports should be acknowledged while distinguishing their anatomy from the complete combination described by Karuru et al. A concise account of the literature considered would help readers understand the basis for the rarity statement. The CARE guidelines encourage authors to place a case in the context of the published literature; they do not require a systematic-review methodology, database screening counts, or a formal systematic search for an individual case report [5]. We therefore suggest wording the claim as “rarely reported” or “an unusual combination” unless a sufficiently comprehensive search supports a stronger assertion.

This is a valuable and well-imaged report of an unusual congenital combination, with a reassuring surgical outcome. Clarification of the relationship between the Doppler-derived TR gradient and the invasively measured RV systolic pressure, including the timing and conditions of the respective measurements, would strengthen the haemodynamic interpretation. Clarification of the jet direction, careful qualification of proposed mechanisms, explicit distinction between angiographic imaging and invasive haemodynamic assessment, and appropriate contextualisation of the rarity claim would further enhance the report’s clinical and educational value.

## Data Availability

No datasets were generated or analysed during the current study.
